# Modelling the effects of environmental conditions on the acoustic occurrence and behaviour of Antarctic blue whales

**DOI:** 10.1371/journal.pone.0172705

**Published:** 2017-02-21

**Authors:** Fannie W. Shabangu, Dawit Yemane, Kathleen M. Stafford, Paul Ensor, Ken P. Findlay

**Affiliations:** 1 Fisheries Management, Department of Agriculture, Forestry and Fisheries, Foreshore, Cape Town, South Africa; 2 Mammal Research Institute Whale Unit, University of Pretoria, Wynberg, South Africa; 3 Applied Physics Laboratory, University of Washington, Seattle, WA, United States of America; 4 Australian Marine Mammal Centre, Australian Antarctic Division, Hobart, TAS, Australia; 5 Centre for Sustainable Oceans Economy, Cape Peninsula University of Technology, Cape Town, South Africa; University of Pavia, ITALY

## Abstract

Harvested to perilously low numbers by commercial whaling during the past century, the large scale response of Antarctic blue whales *Balaenoptera musculus intermedia* to environmental variability is poorly understood. This study uses acoustic data collected from 586 sonobuoys deployed in the austral summers of 1997 through 2009, south of 38°S, coupled with visual observations of blue whales during the IWC SOWER line-transect surveys. The characteristic Z-call and D-call of Antarctic blue whales were detected using an automated detection template and visual verification method. Using a random forest model, we showed the environmental preferences pattern, spatial occurrence and acoustic behaviour of Antarctic blue whales. Distance to the southern boundary of the Antarctic Circumpolar Current (SBACC), latitude and distance from the nearest Antarctic shores were the main geographic predictors of blue whale call occurrence. Satellite-derived sea surface height, sea surface temperature, and productivity (chlorophyll-a) were the most important environmental predictors of blue whale call occurrence. Call rates of D-calls were strongly predicted by the location of the SBACC, latitude and visually detected number of whales in an area while call rates of Z-call were predicted by the SBACC, latitude and longitude. Satellite-derived sea surface height, wind stress, wind direction, water depth, sea surface temperatures, chlorophyll-a and wind speed were important environmental predictors of blue whale call rates in the Southern Ocean. Blue whale call occurrence and call rates varied significantly in response to inter-annual and long term variability of those environmental predictors. Our results identify the response of Antarctic blue whales to inter-annual variability in environmental conditions and highlighted potential suitable habitats for this population. Such emerging knowledge about the acoustic behaviour, environmental and habitat preferences of Antarctic blue whales is important in improving the management and conservation of this highly depleted species.

## I. General introduction

Antarctic blue whales *Balaenoptera musculus intermedia* were widely distributed in the Southern Ocean prior to commercial whaling [1,2,3]. The current population status of Antarctic blue whale remains critically low five decades after the end of whaling; however, there are recent signs of population increase [4,5]. These whales feed predominantly in the euphausiid-rich waters of the Southern Ocean during the austral summer through autumn; and they have been presumed to fast during their over-wintering periods in low latitudes [3,6]. Acoustic monitoring efforts have detected their calls as far north as 8° S in the eastern Pacific and 8° S in the Indian Oceans respectively [7,8].

The very loud (up to ~188 dB re 1uPa @1m) sounds produced by Antarctic blue whales enable passive acoustic survey instruments to detect Antarctic blue whales over considerable distances [9, 10] and thereby investigate the behaviour, ecology, and habitat preferences of these critically endangered whales *in situ* [11, 12]. Antarctic blue whales produce both characteristic three part sounds, known as Z-calls that are frequency modulated from 28 to 17 Hz lasting up to 18 s [13], and D-calls which are variable frequency modulated signals over 106–25 Hz. The Z-calls are produced in long bouts and are thought to be a male reproductive display [13] while D-calls are produced during feeding behaviour by both sexes [14]. Recent detections of blue whale D-calls indicate that some animals are feeding and resident at low latitudes in the Indian Ocean year round [8]. Little is known about the relationship of call rates of Antarctic blue whales and environmental parameters or the effects on environmental variability on blue whale occurrence in the Southern Ocean.

The Southern Ocean is the only ocean basin that is circumpolar; it contains the strong eastward flowing Antarctic Circumpolar Current (ACC) and is the connecting link at all depths for water mass exchanges between the earth's major ocean basins [15,16]. The southern boundary of the ACC is a region of upwelling (i.e. the Antarctic Divergence) that occurs at the Southern Front [17]. Inter-annual variations in the atmospheric pressure at sea level, wind stress, sea surface temperature, cloud cover, and sea-ice extent over the Southern Ocean are important drivers of the ocean circulation in the region [15,18–20]. Annual change in sea ice extent is the major oceanographic/climatic feature of the Southern Ocean and controls the morphological and physiological adaptations of whales, seals, and penguins to life in these frigid waters both through its presence as a physical barrier and as critical habitat for Antarctic krill, *Euphausia superba* [21–23].

Satellite measurements of ocean colour are the principal remote-sensing tool for measuring ocean productivity and its response to climate change/variability (e.g. [18,24]), consequently sea surface chlorophyll-a concentrations (measured as ocean colour) are often used as proxy for primary productivity (e.g. [25]). Remotely sensed environmental parameters have the potential to identify biological hotspots for cetaceans and to therefore establish areas of marine conservation priority (e.g. [21,26,27]).

Here we present the results of analyses on the call type distribution (as call occurrence) and acoustic behaviour (as call rates) of Antarctic blue whales as a function of satellite-derived environmental parameters using the circumpolar passive acoustic measurements during the Southern Ocean Whale and Ecosystem Research (SOWER) program of the International Whaling Commission (IWC), hereafter IWC SOWER. The IWC SOWER program was a long series (1978–2009) of line-transect survey cruises directed primarily at obtaining abundance estimates for Antarctic minke whales [28] and for a period of 14 years (1997–2009) also incorporated a blue whale research component which included collection of acoustics recordings. This paper contains the interpretation of an applied modelling exercise fitting one model to the sets of environmental variables to the observed call occurrence and acoustic behaviour of Antarctic blue whales. Random forest (RF) model reveals the response of Antarctic blue whales to environmental variability and also highlight the importance of environmental variability in monitoring the status of Antarctic blue whales.

## II. Acoustic data

### Developing and testing detectors

**Fig 1:**
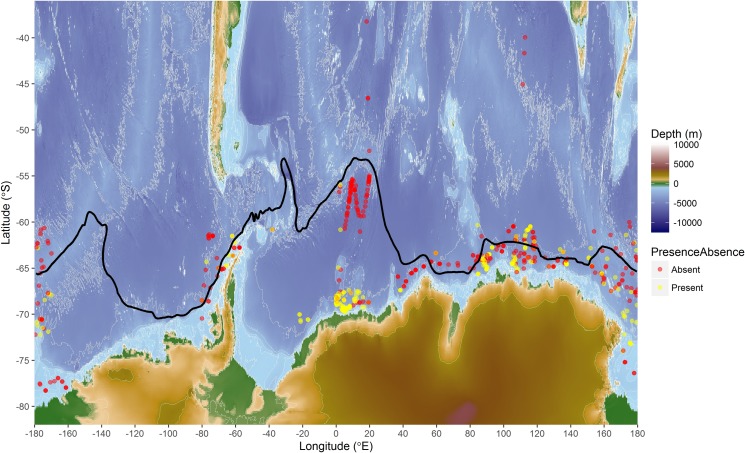
Map showing the location of sonobuoy deployments. Those with acoustic detections of Antarctic blue whales are shown as yellow dots. Locations with no blue whale detections are shown as red dots. The black line represents the positions of SBACC based on Orsi et al. [31].

**Fig 2:**
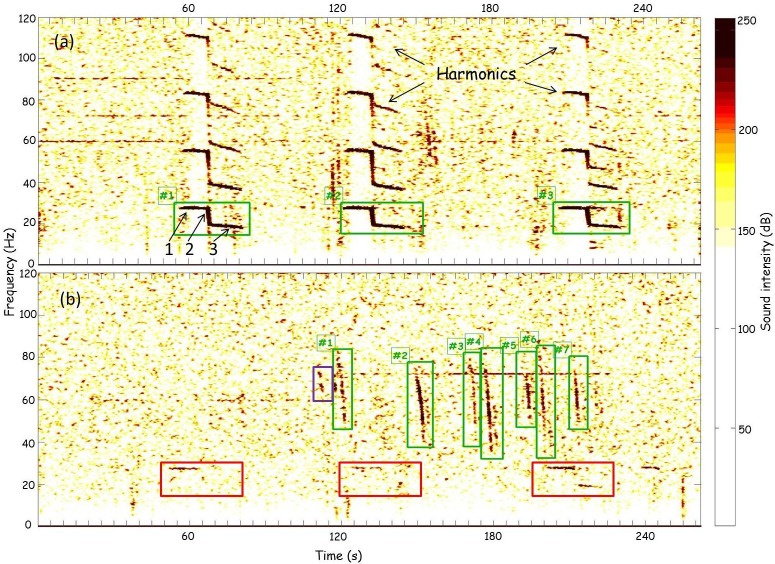
Spectrograms showing the two types of Antarctic blue calls; a) the three-unit (1, 2, and 3) frequency modulated 28 to 17 Hz blue whale Z-calls (green rectangles), and b) high frequency 90–30 Hz downsweeping D-calls (green rectangles). Each green rectangle represents calls detected by the detector and the hash (#) represents the count of each call detected. Also presented are one-unit faint Z-calls (red rectangles) and faint D-call (purple rectangle). Spectrogram parameters: frame size 1.28 s, 25% overlap, FFT size 4096 points, Hanning window.

We analysed passive acoustic data collected using both sonobuoys and towed hydrophones during the IWC SOWER cruises conducted south of 55°S to the ice edge from December to February of 1996/97 to 2008/09. Some acoustic observations extended as far north as 38°S during transits to and from the Antarctic (Fig 1). Acoustic data analyses were performed in the eXtensible Bio-Acoustic Tool (XBAT) software [29] implemented as a MATLAB routine [30] with automatic detection templates for Z- and D-calls developed and applied in XBAT. A complete Z-call with all the three-units (Fig 2A) and the D-call downsweeping from 90–30 Hz (Fig 2B) were used as templates because they both contained most of the energy of the calls. The first unit (8–12 s) of the Z-call is at 28 Hz (Fig 2A1), the second unit is relatively short (2–5 s) and downsweeps from 28 to 19 Hz (Fig 2A2), and the third unit (8–12 s) is a slightly frequency-modulated between 19 and 17 Hz (Fig 2A3). Multi-harmonics of Z-calls (Fig 2A) reflect the strong sound energy level at the receiver usually associated with that sounds produced by animals closer to the recorder whilst faint Z-calls with weak energy levels are those produced by animals farther from the recorder (Fig 2B).

The template detector method operates on an acoustic time series of spectrograms by constructing a correlation kernel for the vocalization [32]. Calls were recognized from spectrograms by cross-correlating with the template kernel based on a similarity level above a set threshold. We used effective detection templates from 1997 to automatically detect Z- and D-calls from 1997 to 2004 acoustic data, and then templates from 2009 were used for 2006 to 2009 data to account for changes in fundamental frequency of the Z-call [33]. In order to assess the performance of the automated detectors, the entire acoustic dataset (1,518 hours) was assessed visually to estimate the number of false positive (detections that were not blue whale calls) and false negative (missed blue whale calls) calls. The visually identified false positive detections were manually excluded from further acoustic analyses. Visually identified false negatives were manually incorporated into the final total call number calculations.

### Outcomes of detectors

**Fig 3:**
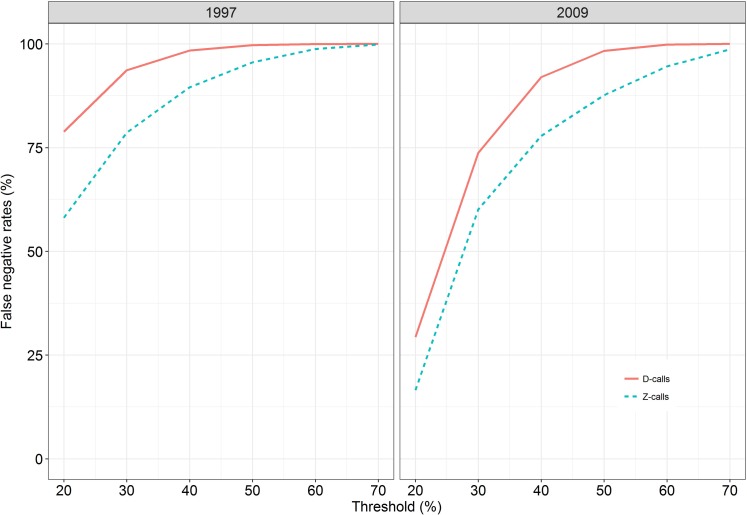
False negative rates at different thresholds for Z- and D-calls using the 1997 (left) and 2009 (right) detection templates.

We tested six different thresholds from 20% to 70% by increments of 10% to determine optimal thresholds for our analyses (Fig 3). The 20% detection threshold was best suited to our study as it produced the fewest missed calls compared to other thresholds, although the number of false positives was much higher. Low thresholds are generally known to be effective at scanning through large data set such as the one used here [32]. False negative error rate refers to the percentage of false negative calls. Detection templates from 1997 produced false negative error rates of 58% for Z-calls and 79% for D-calls; and accuracy rates of 42% for Z-calls and 21% for D-calls (Fig 3). On the other hand, templates from 2009 produced false negative error rates of 17% for Z-calls and 29% for D-calls; and accuracy rates of 83% for Z-calls and 71% for D-calls (Fig 3).

### Detector caveats

Automated detection of animal vocalizations is effective at accelerating acoustic data analyses and producing more robust results than human visual sound detections [32,34]. The very high false negative error rates and low accuracy rates of the automatic detector produced by the 1997 templates could indicate the identified change of blue whale call frequency shift over time [33,34]. In contrast, the 2009 templates analysed data within relatively comparable time periods, hence produced relatively better detection performances. The fact that not all recorded Z-calls contained the three-units of the complete call used in the template and also that not all recorded D-calls contained frequency downsweep range of the detector (Fig 2) could have induced challenges to this automatic cross-correlation detection method. Noise, possibly from airguns and other unknown sources, contributed false positive error rates for both call types. Overall, the XBAT template detector algorithm was useful at detecting highly stereotyped blue whale calls even though it yielded higher false negative rates and lower accuracy rates than other studies (e.g. [32–34]). Nonetheless, the method could be improved by an allowance to simultaneously use of more than one call template. For example, the simultaneous use of 3 templates for the Z-calls, i.e. one complete three-unit call template, plus a two-unit call template and one-unit call template (Fig 2) and/or the use of templates from different years simultaneously. Such multi-template will permit the use of high thresholds without inducing high false negative error rates.

Širović [34] observed that intra- and inter-annual changes of call frequency affected the automatic detection of Northeast Pacific blue whale *B*. *m*. *musculus* calls, and recommended a new detection template for each year. More detailed estimates of the changes of start and end frequency of Antarctic blue whale calls over time from this SOWER acoustic dataset and the most recently recorded blue whale calls both in the Southern Ocean and low latitudes will inform on the long term behavioural changes in blue whale vocalizations and whether such templates for each year should be used to reduce false detections.

### Whale numbers and groups

Acoustic stations (484 total) were differentiated into two types: type 1 stations were sonobuoy deployments in association with a visual sighting of blue whales (107 stations); type 2 stations were deployments without blue whales visually detected (377 stations). Estimates of numbers of individual blue whales and of blue whale groups observed in type 1 acoustic stations were sourced from the acoustic data forms completed during the IWC SOWER cruises as provided by the IWC Secretariat and from the line transect visual survey sighting forms, if whales were seen during the normal line transect visual surveys. The date, time of the day, and station number of each acoustic recording were used to link blue whale sightings to the acoustic results. In instances where blue whale calls were detected from the analyses but not heard during the *in situ* monitoring, the whale numbers and number of groups seen were assumed to be zero unless a matching time and position with a whale was found in the visual sighting forms.

### Call rate calculation

The number of calls recorded from each sonobuoy and/or hydrophone deployment were allocated to each station as documented in the IWC SOWER cruise reports and acoustic forms. The recording duration of each deployment was determined during acoustic data analyses, and the station duration was calculated as the sum of durations of recordings from that station. Call rates of both the Z- and D-calls were calculated for each station as the total number of calls divided by the station duration to give calls per hour at each station. Call rates were not determined as calls per hour per individual, however, but as call rates per group(s) per hour due to the difficulty in determining the precise number of vocally active animals within any groups. Results of call rates were thereafter assigned into day (06:00 to 18:00 local time) and night (18:00 to 06:00) depending on their recording time before fitting to the models, although such day/night differentiation reflected the survey mode of the vessel rather than Antarctic summer light regimes as visual monitoring only took place from 06:00–18:00 each day.

## III. Modeling blue whale occurrence and behaviour

### Environmental data used

Satellite chlorophyll-a (chl-a) measurements were obtained from the globally blended and binned high resolution level-3, case 1 water GlobColour project [35] that merges the remotely sensed ocean colour measurements from three satellite data sources: MERIS (Medium Resolution Imaging Spectrometer), MODIS-Aqua (Moderate-resolution Imaging Spectroradiometer) and SeaWiFS (Sea-viewing Wide Field-of-View Sensor). However, only the SeaWiFS measured global chlorophyll-a data were available for December 1997 through February 2002. The GlobColour chlorophyll-a data were extracted at a monthly temporal resolution and 0.05° (approximately 4 km) spatial resolution in the regions of the South Ocean surveyed during the IWC SOWER cruises. Three month chl-a averages were calculated for each surveyed resolution point where chl-a values were not available due to cloud cover. All three months used in this study fell within the same summer season associated with sustained phytoplankton blooms (e.g. [26]). Thus, the December 1997 average chl-a concentrations were used for January and February 1997 because there were no chl-a data for those months as the GlobColour chl-a time series only began in September 1997. Data were processed in R statistical software package [36] using the following packages: Matrix [37], stringr [38], and ncdf4 [39]. The isin.convert.R function from www.menugget.blogspot.com was used to convert the binned GlobColour chl-a data to a grid format.

Satellite-derived sea surface temperatures (SST) were obtained from the Group for High Resolution Sea Surface Temperature (GHRSST) global Level 4 sea surface temperature analysis produced daily on a 0.25° grid spatial resolution at the NOAA National Climatic Data Center [40]. GHRSST product uses optimal interpolation (OI) boosted by data from the 4 km Advanced Very High Resolution Radiometer (AVHRR) Pathfinder Version 5 time series (when available, otherwise operational NOAA AVHRR data are used) and *in situ* ship and buoy observations. The OI analysis is a daily average SST that is bias adjusted using a spatially smoothed 7-day *in situ* SST average and is therefore tuned to about 0.3 meter [41]. The Open-source Project for a Network Data Access Protocol (OPeNDAP) facility was used to get SST data via the Environmental Research Division's Data Access Program (ERDDAP) data server using the rerddap [42] and xtractomatic [43] packages in R as a simple, consistent method to download subsets of data from a gridded dataset via a specially formed Uniform Resource Locator (URL). The daily SST products were subsequently averaged into monthly SST products.

Sea surface height (SSH) daily measurements were obtained from the Archiving, Validation and Interpretation of Satellite Oceanographic (AVISO) data program using the erdTAssh1day function in R. AVISO SSH product combines data from up to six multiple satellites, including Jason-1, TOPEX/Poseidon, the European Remote Sensing (ERS) Satellites 1 and 2 and their successor, the Environmental Satellite (ENVISAT), and the Geodetic Satellite (GEOSAT) [44]. The monthly SSH was subsequently computed from the daily product. The Southern Ocean bathymetry data (Fig 1) were obtained from the ETOPO1 global relief model that integrates land topography and ocean bathymetry using the Earth's surface 1 arc-minute global relief model [45].

Monthly blended vector sea surface wind speed and direction (at 10 m above sea level) and sea surface wind stresses were downloaded from ftp://eclipse.ncdc.noaa.gov/pub/seawinds/SI/ and processed in R using custom built functions. These wind speeds and directions are blended from multiple satellites (up to six, including the DMSP Special Sensor Microwave/Imager (SSM/I), the Tropical Rainfall Measuring Mission (TRMM), Microwave Imager (TMI), the Quick Scatterometer (QuikSCAT) and the Advanced Microwave Scanning Radiometer-EOS (AMSR-E)) observations, on a global 0.25 degree spatial grid and a 6-hourly temporal resolution (see [46]). The sea surface wind stress, tau (τ), was used for our modelling since it is the wind stress amplitude as a scalar mean given in N m^-2^. Distance in kilometres (km) to the nearest Antarctic coastline was calculated using the custom developed function defined as the shortest distance to the Antarctic coastline for each of the acoustic stations.

Monthly sea ice extents were downloaded from the G02135 dataset [47] at the National Snow and Ice Data Centre (NSIDC) data pool repository: ftp://sidads.colorado.edu/DATASETS/NOAA/G02135/shapefiles/. Sea ice index version 2 uses V1.1 of the sea ice concentrations from Nimbus-7 SMMR and DMSP SSM/I-SSMIS Passive Microwave Data (also known as GSFC product) as the input data source for the final portion of the Sea Ice Index record. The data are a level-3 gridded product, mapped to a polar stereographic grid at a spatial resolution of 25 km. Data were processed using the rgdal mapping package [48] in R. Distances (km) of acoustic stations to the ice edge were calculated.

We used positions of the southern boundary of ACC (Fig 1) defined by Orsi et al. [31] using historical hydrographic data from the Southern Ocean. The data with the positions were downloaded from http://gcmd.nasa.gov/records/AADC_southern_ocean_fronts.html. The distance (km) of acoustic stations to the closest southern boundary of ACC calculated using a custom design R function.

### Model choice

Before modelling the data, we determined the effects of multi-collinearity between predictor variables using the generalised variance inflation factors (GVIF; [49]) implemented through the car library [50] in R. GVIF is used to describe how much multicollinearity (correlation between predictors) exists in regression analyses such as Random Forest (RF). Low GVIF values (around one) indicate weak or no correlations, GVIF values around five indicate moderate correlations; and values of 10 or more indicate strong correlations [51,52]. Our GVIF values ranged from 1.1 to 5.8 when excluding distance to the ice edge but reach a maximum of 8.9 when including distance to the ice edge, thus distance to the ice edge was eliminated as it is highly correlated with latitude and SST and its GVIF value was close to the elimination threshold of 10 [52].

RF models are known to provide higher performance and have a number of advantages over standard regression methods like generalized additive models [53–55]. RF uses a set of unpruned decision trees that are bootstrapped as they grow with trained sample data, and rely on randomly chosen subsets of the predictor variables as candidate splitting tree nodes [55,56]. RF does not discount some variables completely and candidate split-variable selection increases the probability of any solitary variable being included [55,56]. A generalised boosted regression trees model (GBM) was also used to model the occurrence and acoustic behaviour of blue whales but RF was found to be better at detecting signals and had a higher prediction accuracy than GBM.

RF model was used to determine which environmental parameters influenced the acoustic occurrence (presence/absence of calls) and acoustic behaviour (call rates) of the Antarctic blue whales during the Southern Ocean summer. The chl-a data were log-transformed before model fitting due to the skewness of their distribution. Pearson’s correlation coefficients (r) were calculated to measure the linear correlation (dependence) between the call rate of each call type.

The relative importance of predictor variables in the model was assessed by computing the influence of each of the variables on the prediction error of the model. The relative importance of each of the variables in the model can be computed by permuting the Out Of the Bag (OOB)/test data sample or by determining the decrease in node impurity, as measured by the mean sum of squares, resulting from splitting of the variable of interest and averaging over all trees. For each tree the prediction error is computed on the OOB data and the permuted data are calculated and averaged across all trees and normalized by the standard deviation of the difference. This normalized index is calculated for each of the variables and used as index of relative importance. RF model was fitted to determine the importance of the following predictors on blue whale occurrence: longitude, latitude, log-transformed chl-a, SST, SSH, water depth, distance from the shoreline, distance from the nearest SBACC, wind stress, wind speed, wind direction, station duration, day/night, and month. RF model for call rates determined the importance of the following predictors: longitude, latitude, log-transformed chl-a, SST, SSH, water depth, distance from the shoreline, distance from the nearest SBACC, wind stress, wind speed, wind direction, station type, whale numbers, day/night, month and whale groups.

The RF optimal parameter configurations for both call rate models were: the number of growing trees ntrees = 500 for Z-calls and 3,000 for D-calls; the splitting minimum size of terminal nodes of trees nodesize = 2 for Z-calls and 1 for D-calls; and the number of call rates randomly selected at tree node mtry = 3 for Z-calls and 2 for D-calls. The RF optimal parameter configurations for blue whale occurrence modelling: ntree = 500, nodesize = 1, and mtry = 6.

Since the detection ranges of sonobuoys were not estimated here, we ran the RF model with and without sighting data using the above optimal parameter configurations to evaluate effects of detection ranges on blue whale call rates. The model produced relatively similar sets of outputs in both cases. The modelling was performed with the R statistical software package using libraries randomForest [57] and ranger [58].

We used the 5-fold cross-validation (5-foldCV) and the Leave Group Out Cross Validation (LGOCV, also known as Monte-Carlo cross validation), to quantify the predictive accuracy of our classification model type between the predicted values and observed values of blue whale occurrence [59]. The area under the receiver operating characteristic curve (AUC) was used to measure the predictive accuracy of our classification model, i.e. how well the model correctly classified the classes included in the model (here the presence/absence of call whales). AUC normally takes values between 0.5 and 1, where values closer to 1 indicate better classification ability. The number of replicates (nrep) was set to 200 for the model performance assessment.

Using 70% of the data for training and the remaining for validation, the performance of RF for call rate modelling was assessed. Root mean square prediction error (RMSPE) was used to measure the difference between values predicted by the model and observed values. In addition, Spearman's rank correlation coefficient (rho) was applied as a qualitative measure of the performance of the model. A low RMSPE value indicates a better model whereas the opposite is true for rho.

The AUC values indicated better performance for RF than GBM (Table 1). The correlations between the predicted and observed values of both call rates were also highest for RF (Table 2). The 5-fold cross validation and LGOCV broadly produced remarkably similar results (Tables 1 and 2).

**Table 1:** RF model performance for blue whale occurrence based on AUC.

TypePerf	Mean	SD
5-foldCV	0.957	0.004
LGOCV	0.957	0.008

TypePerf is the type of performance and SD is the standard deviation.

**Table 2:** RF model performance for blue whale call rates based on RMSPE and Spearmen’s rho.

		rho		RMSPE	
Call type	TypePerf	Mean	SD	Mean	SD
D	5-foldCV	0.647	0.012	23.567	0.754
D	LGOCV	0.645	0.026	23.425	1.663
Z	5-foldCV	0.842	0.009	8.651	0.483
Z	LGOCV	0.841	0.018	8.722	0.872

### Southern ocean environmental conditions

**Fig 4:**
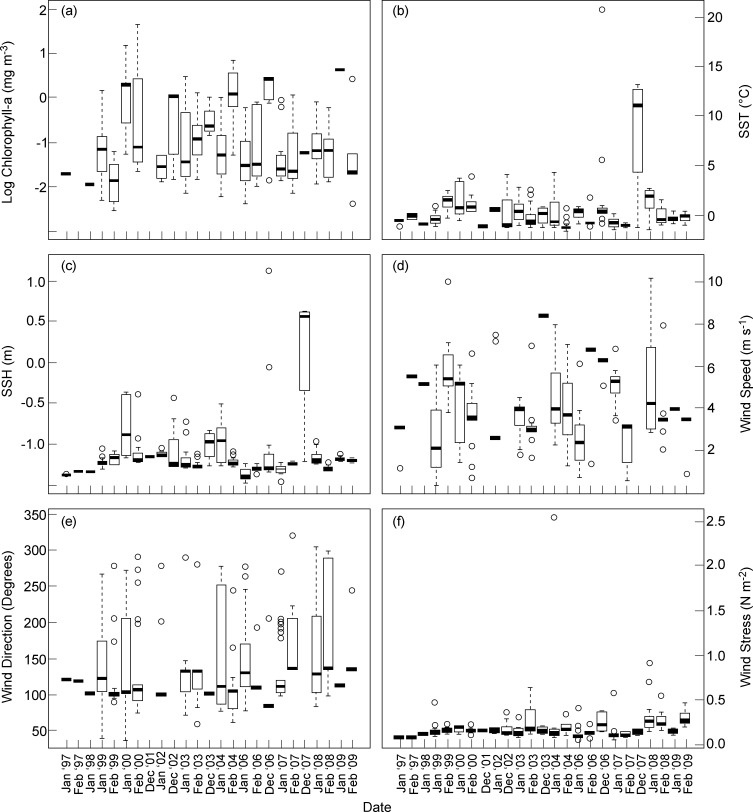
Monthly variations of the six satellite-derived environmental predictors used in models for the summer (December–February) of 1997 to 2009 in areas surveyed by the IWC SOWER cruises in the Southern Ocean (38–78°S, -180-180°E). (a) Log transformed blended chl-a concentrations (mg m^-3^), (b) Sea surface temperatures (°C), (c) Sea surface heights (m), (d) Wind speed (m s^-1^), (e) Wind direction (degrees), and (f) Wind stress (N m^-2^). Note that not all environmental variables were acquired for all months of our study period due to satellite data unavailability.

Southern Ocean environmental parameters varied across the 14 years of the IWC SOWER cruises (Fig 4), although it must be noted that different regions were normally visited in different years. The blended log-transformed chl-a concentrations for all surveyed years ranged between 2.5 and 1.7 mg m^-3^ (equivalent to 0.1 and 5.3 mg m^-3^ untransformed chl-a concentations; Fig 4A). The highest chl-a concentration was found at 62.78°S and 57.65°W for a station from February 2000 whilst the lowest was at 62.58°S and 119.28°E for a station from February 1999. Chl-a concentrations were generally higher for December and February between years (Fig 4A). The highest SST value of 20.6°C was derived in the Atlantic Ocean at 38.25°S and 18.29°E during the 2005/2006 cruise and the lowest recorded SST of -1.6°C was off the ice edge at 63.55°S and 64.4°W during 1999/2000 cruise (Fig 4B).

**Fig 5:**
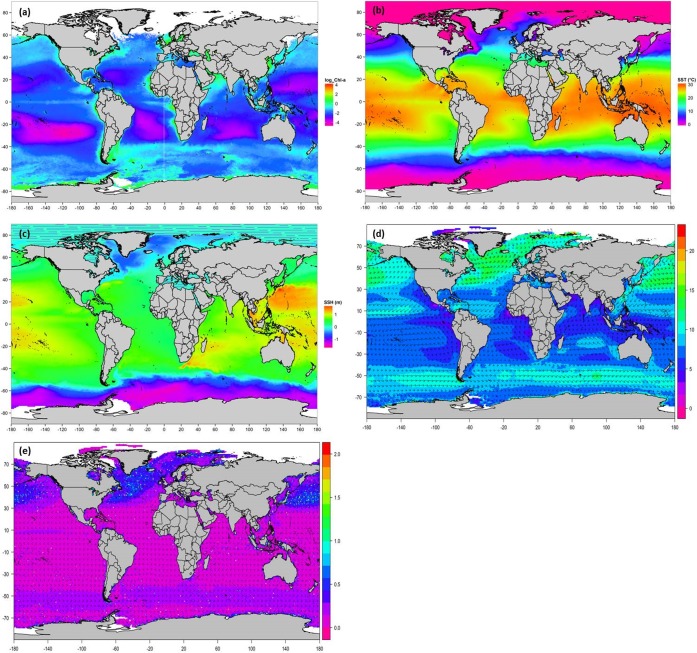
Global climatology of environmental variables observed during the austral summers (December- February) of 1997–2009. Horizontal distribution of the (a) log-transformed chl-a, (b) SST, (c) SSH, (d) sea surface wind speed (color) in m s^-1^ and vector (arrows), (e) sea surface wind stress (color) in N m^-2^ and vector (arrows). Only SeaWifs chl-a data were used for plotting the relative distribution of chl-a concentrations.

Sea surface height was generally below -1 m with December and January having higher SSH values (Fig 4C). Wind speeds fluctuated across all years (Fig 4D). Wind direction medians ranged between 100° and 150° (Fig 4E). Wind stress medians for all the years were well below 0.4 N m^-2^ (Fig 4F). January 2006 had the highest value of 2.5 N m^-2^ wind stress observed at 62.75°S and 178.89°W that corresponds to wind speeds of 70 kts we observed during that IWC SOWER cruise. The year 2007/08 had environmental anomalies where SST, SSH, wind speed, wind direction and wind stress were higher than previous years (Fig 4). The distributions of the main six satellite-derived environmental variables across the data distribution in the Southern Ocean show both latitudinal and longitudinal trends over the study period (Fig 5).

### Blue whale call rates

**Fig 6:**
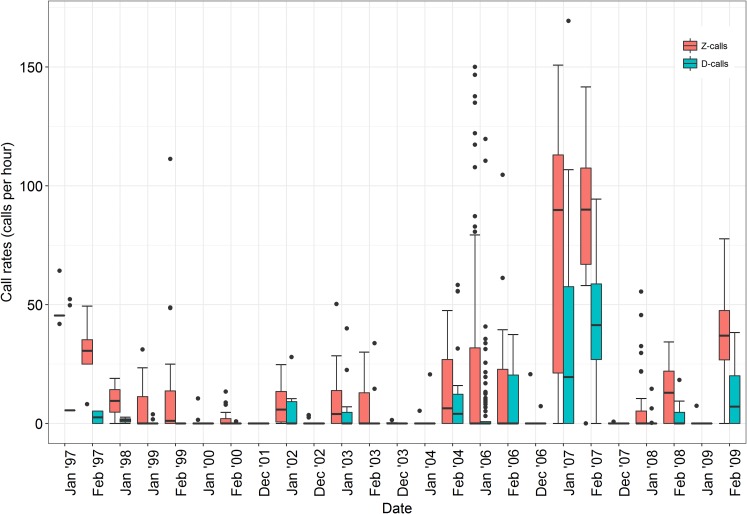
Box and whisker plots showing the Antarctic blue whales vocalization rate trends for the Z- and D- calls detected over the summer months during the IWC SOWER surveys. The box represent the first quartile to the third quartile (the interquartile range), and the segment inside the box is the median. The whisker delineates 1.5 times the interquartile width, and the closed circles are observations that are outside the range covered by the whisker.

Vocalisation or call rates are important for determining the approximate acoustic behaviour of whales at a given time and place [60]. Type 1 stations included 375 blue whales sighted in 173 groups associated with the call rates, and call occurrence, from 484 acoustic stations. Antarctic blue whale Z- and D-call rates generally increased between January and February of each year (Fig 6). Median D-call vocalisation rates for stations during most IWC SOWER years were well below 10 calls per hour (Fig 6). Blue whale call rates for both call types were generally low during December months for all the years. D-call rates for all the IWC SOWER cruises were observed to be highly correlated (r = 0.68) with the Z-call rates.

### Acoustically detected whale occurrence modelling outputs

**Fig 7:**
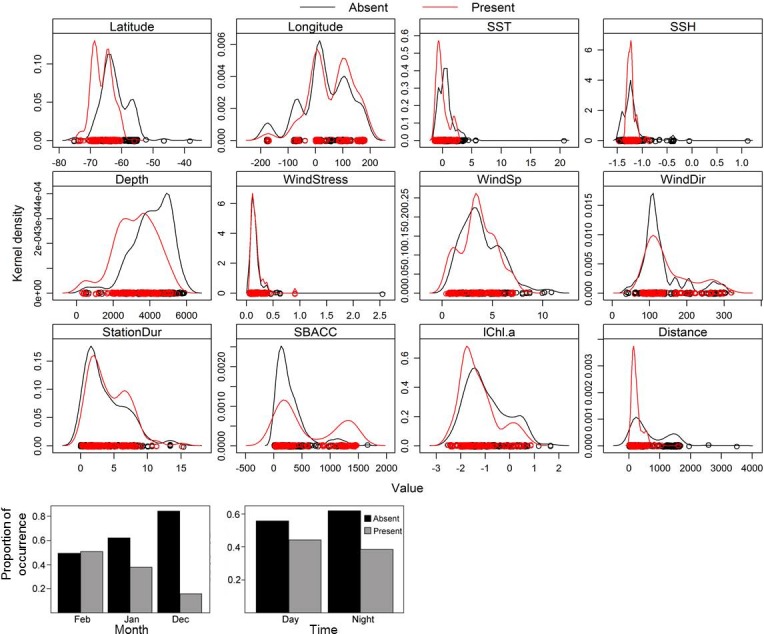
Kernel density distribution of each of the variables used in this study to define the occurrence of both blue whale calls in the Southern Ocean. Open circles represent each presence and absence of blue whales while barplots are for factor variables. SBACC is the distance of acoustic station from the nearest southern boundary of ACC (km), StationDur is station duration (hrs), lChl.a is log chlorophyll-a (mg m^-3^), Distance is the distance from the nearest Antarctic shore (km), WindSp is wind speed (m s^-1^), and WindDir is wind direction (°).

**Fig 8:**
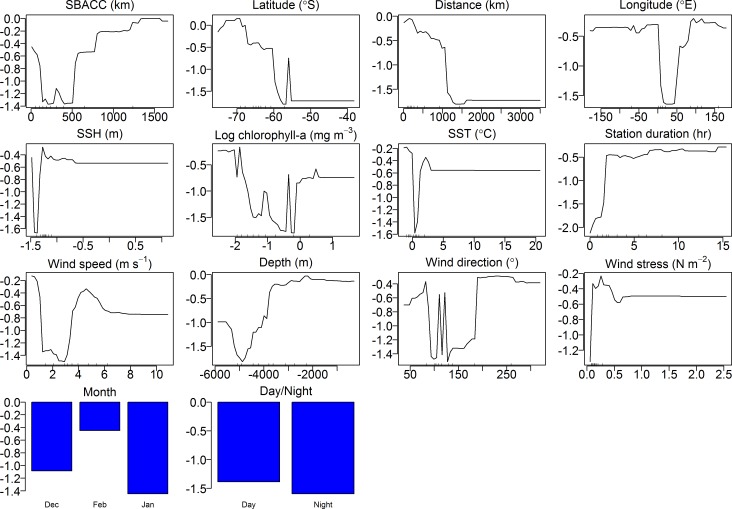
RF model plots of partial effects of the different predictors on the occurrence of blue whale D- and Z-calls. Y-axis is the partial effect of each predictor on occurrence (in logit-scale).

**Fig 9:**
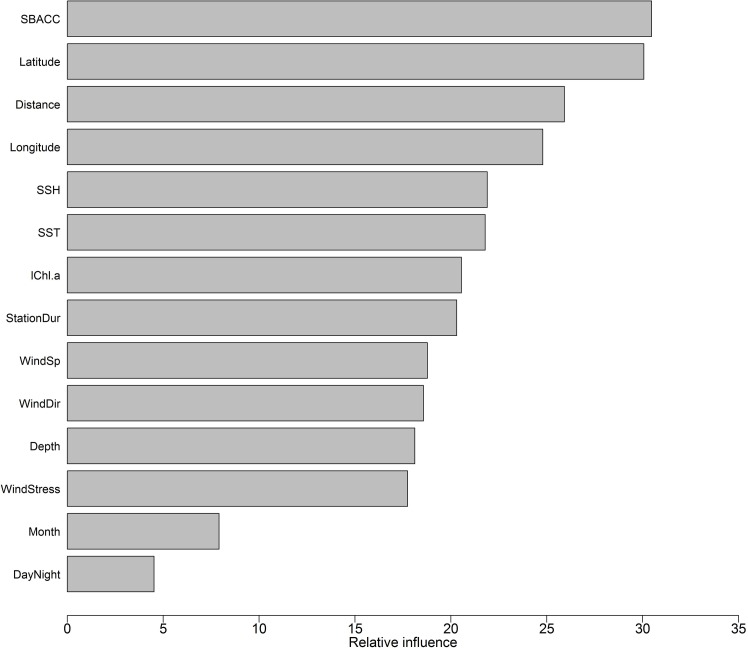
Relative importance of the different predictors influencing the occurrence of blue whales based on RF. Relative variable importance is measured by residual sum of squares and expressed relative to the maximum.

Presence or absence of a species (Fig 1) can be used to determine preferred habitat, and the response curves of the effects of different environmental parameters on the occurrence of blue whales show such preferences. Blue whale call presence showed variation relative to different predictor parameters (Fig 7). The effects of each of the parameters on the occurrence of blue whale calls are shown on the response curves in Fig 8. Distance from nearest SBACC, latitude,distance from the nearest Antarctic shores and longitude, were the most important predictors of blue whale occurrence (Fig 9). SSH, SST, log chl-a, station duration, wind speed, wind direction, depth, and wind stress were moderately important predictors of occurrence, whereas month and day/night were the least important predictors of occurrence (Fig 9). Blue whales were only heard in water depths between 308 and 5,888 m.

### Acoustic behaviour modelling outputs

**Fig 10:**
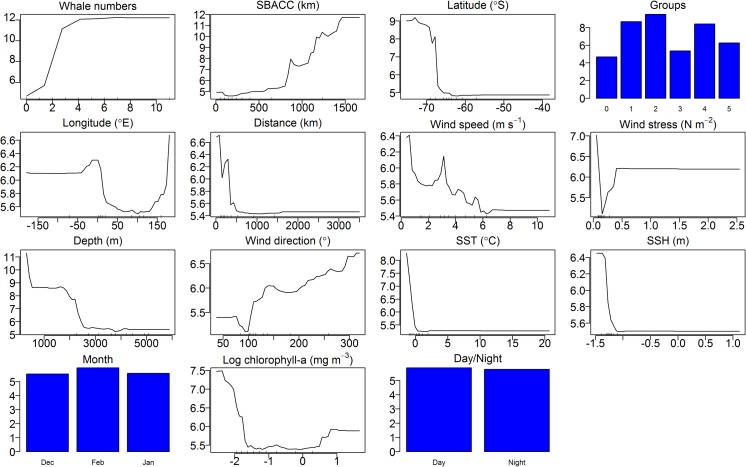
Partial effects of the different predictors on D-call rates of blue whales using the RF model. Plots indicate the marginal effect on blue whale call occurrence (y-axes) by each predictor variable (x-axis).

**Fig 11:**
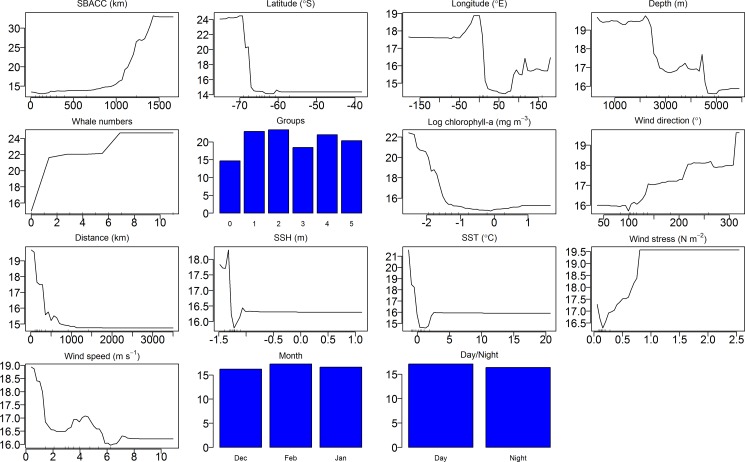
Partial effects of the different predictors on Z-call rates of blue whales using the RF model. Plots indicate the marginal effect on blue whale call occurrence (y-axes) by each predictor variable (x-axis). Contribution of each variable to the model given below the function. Y-axes are different across all plots. Scale of x-axes is different across each predictor variable.

**Fig 12:**
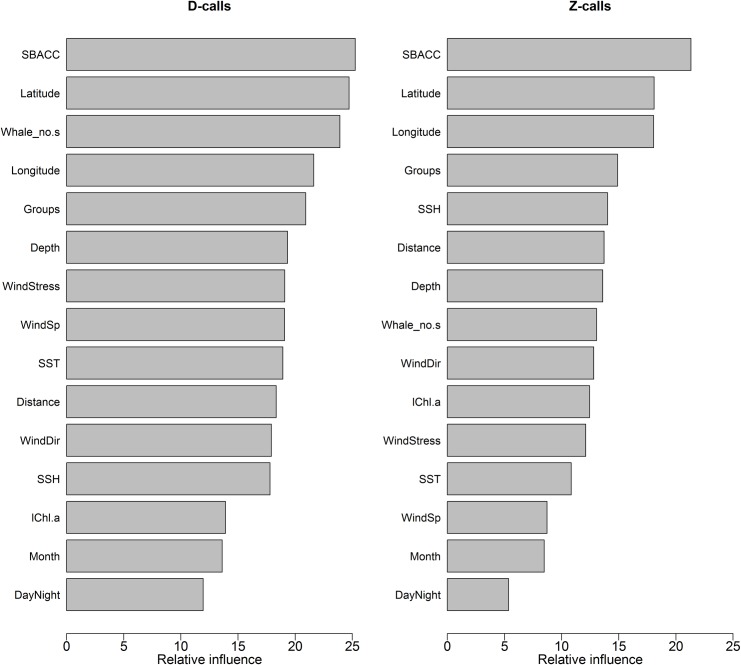
Relative importance of the different predictors in influencing the acoustic behaviour of blue whales based on RF model results. Relative variable importance is measured by residual sum of squares and expressed relative to the maximum.

Model response curves of the effects of 15 different summer environmental parameters on the call rates of the two blue whale call types based on the 14 years acoustic time series are shown in Figs 10 and 11. The RF model found that the distance from the SBACC, latitude and number of whales at a station were the most important predictors of D-call vocalisation rates (Fig 12). Longitude, followed by whale groups (number of group sizes of 1, 2 and 4 were mostly influential), depth, wind stress, wind speed, SST, the distance from the Antarctic shore, wind direction, and SSH were moderately important predictors of acoustic behaviour (Fig 12). Log chl-a, month and time of day were the least important predictors of D-call rates (Fig 12). Distance from the SBACC, latitude and longitude were the most important predictors of the blue whale Z-call vocalisation rate by the RF model (Fig 12). Whale groups (number of group sizes of 1, 2 and 4 were mostly influential) followed by SSH, the distance from the Antarctic shore, depth, number of whales, wind direction, log chl-a, wind stress, and SST were the moderately important predictors of behaviour (Fig 12). Wind speed, month and time of day were the least important predictors of Z-call rates.

## IV. Blue whales in the Antarctic environment

Environmental conditions were important in predicting the call occurrence and acoustic behaviour of blue whales in the Southern Ocean. Call rates increased in January and February of each year, likely due to the increasing numbers of blue whales arriving in the Southern Ocean from their overwintering grounds relative to December where more extensive sea ice extent could potentially have affected the distribution of vocal Antarctic blue whales [61,62]. However, month of survey was the least important predictor of both call occurrence and vocalisation rates in our modelling outputs. We suspect that this is because the survey months of all IWC-SOWER cruises fall within the same season, hence their overall results are comparable although there might be some inter-month differences.

Not surprisingly, the type 1 (presence of whale group ≥1) station had a higher effect on call rates than type 2 (no whale groups present) station (Figs 10 and 11), confirming that deploying an acoustic instrument in the visual presence of whales increased the probabilities of recording their sounds, and that there might be other unobserved vocally active whales in the area interacting with the sighted whales. Acoustic stations where blue whales were not detected might indicate that whales were absent from the area or silent. Call rates increased with number of whales and groups in an area, a behaviour also noted in North Atlantic right whales *Eubalaena glacialis* [60]. The recording duration of stations was found to be a moderately important predictor of the call occurrence of blue whales by the RF model, reflecting that the length of the acoustic recording affects the probability of detecting blue whales within an area [13]. Although higher call rates were recorded during the day than at night, time of the day was the least important predictor because acoustic occurrence and vocalisations occurred independently of time of the day.

In this study, Antarctic blue whales were commonly found in waters with SSTs between -1.4°C and 3.5°C, which suggests that this species has a broad temperature tolerance in the Southern Ocean. This could also explain the latitudinal dependence of blue whales, as the colder waters are generally found further south near the ice edge. D-call rates increased when SSTs were below 0.3°C illustrating a preference for temperatures associated with high zooplankton productivity of the ACC thermal fronts. Inter-annual variability in SST and mesoscale ocean circulation affect the inherent Antarctic ice cover that is critical to the distribution of blue whale prey species *Euphausia superba* [15,18]. SST values between 0–3.5°C were demonstrated by the RF model as most preferred range for the Z-call rates.

The area between the ice edge and 800 km from the nearest Antarctic shore was determined by the model to be important for call occurrence and call rates of Antarctic blue whales in summer. This indicates that not all blue whales transit to the ice edge to feed, but that some animals remain well north of the ice edge. The SBACC is associated with high primary production, krill and whales, suggesting that it provides predictably productive foraging for many species; it is of critical importance to the function of the Southern Ocean ecosystem [63,64]. However, our results showed that regions further away from the SBACC but closer to the ice edge, were the most preferred by blue whales. This result shows that blue whales in the Southern Ocean prefer productive areas closer to the Antarctic shores.

Water depths between 380 and 5,900 m at the ice edge and open sea, respectively, were of importance for both the occurrence and acoustic behaviour of whales reflecting a wide bathymetric preference by the species. It is important to note that the location of the ice edge in summer generally approximates the continental slope front (i.e. the shallower isobaths in this study) and is normally a region of high chl-a (Fig 7A) and prey availability. Kasamatsu et al. [65] also found that blue whales preferred the ice edge regions. Latitudes between 60°S and 75°S were important for the occurrence and vocalisation rates of blue whales. Longitudes between 50°W and 30°E, and 90°E and 180°E were important longitudinal bands for the blue whale occurrence and vocalisation rates reflecting that these areas are associated with bathymetric features that are important for phytoplankton blooms in the Southern Ocean [16]. Not surprisingly, more devoted survey effort in these longitudinal bands resulted in higher probability of encountering blue whales [13]. High historical blue whale catches recorded in these longitudinal bands confirm these areas as preferred habitats for blue whales in the Southern Ocean [3].

SSH values of less than -1 m were important to blue whale occurrence and call rates, indicating that blue whales occurred closer to the Antarctic Peninsula as SSH decreased closer to the Antarctic Peninsula [66]. Specific values of SSH are known to define fronts and eddies in the Southern Ocean which in turn increase prey abundance or availability by enhancing primary production [17,67]. Sea surface wind stress is considered important in the Ekman’s transport of the wind-driven currents such as the ACC by leading to upwelling and downwelling in different areas of the ocean [16,17,68]. The satellite-derived wind stress values of this study were within the 0.5–1.0 N m^-2^ range observed by Nowlin and Klinck [17] and Thomalla et al. [69] for the Southern Ocean, although Nowlin and Klinck [17] observed wind stress values around 2.5 N m^-2^ between 20°W and 110°E. Wind direction controls the supply of nutrient carrying sediments to the continental margin, thus is an important sign of ocean productivity which is closely coupled to climate variability [18]. The occurrence and vocalisation rates of blue whales showed strong preferences for south-easterly to north-westerly winds that are consistent with winds away from, and returning to, the Antarctic continent suggesting evidence of high productivity associated with those wind directions.

Satellite-derived sea surface wind speeds of less than 10 m s^-1^ were experienced across the survey regions, and these wind speeds were typical for the Southern Ocean [46,70]. These wind speeds coincide with the good to moderately good weather conditions required to conduct line transect visual surveys and sonobuoy deployments. Furthermore, such wind speeds are important for ocean circulation, air-sea gas and chemical exchanges and nutrient transport (e.g. [16,17]) and are generally indicative of phytoplankton productive areas associated with fronts and eddies. Wind speed is also important acoustically in the generation of oceanic underwater noise [71], and may influence the acoustic behaviour of marine mammals [72]. Our RF results showed that detections of vocalisation by blue whales were higher at wind speeds below 6 m s^-1^ (Figs 10 and 11).

Deployments of acoustic instruments in poor weather conditions (at wind speeds above 10 m s^-1^) are needed to further predict the temporal and spatial effects of wind-induced noise on the acoustic behaviour of blue whales (although wind noise might impact the detection of calls). High wind speeds are also known to introduce air bubbles in the upper water column that absorb and refract sound [73]. The uses of stationary or moored hydrophone such as autonomous acoustic recorders or mobile ocean gliders to record animal sounds have the potential to reveal the possible effects of wind on the acoustic behaviour of blue whales at a high spatial and temporal resolution. This can also provide some information on whether eddies are able to induce high chl-a that attract blue whales. As these dynamic instruments (i.e. gliders) are capable of being controlled remotely, they have the potential to follow a particular feature of interest such as a meandering front or eddy.

Available evidence demonstrate that krill are most abundant in areas with the highest-productivity within their habitats (e.g. [74]), yet simplistic correlations have not been found between productivity in the Southern Ocean and krill abundance [75,76]. Negative krill-phytoplankton relationships found in the Southern Ocean may reflect locally high krill densities that drive down the phytoplankton biomass [75,77], thus high krill densities (the major phytoplankton consumer in the Southern Ocean) can be associated with locally low chl-a concentrations. Considerable variation in chl-a concentration was observed among different years and locations across the survey area, and there was a negative relationship between the acoustic occurrence of blue whales and chl-a concentrations. Branch et al. [3] and Širović and Hildebrand [78] also observed a negative relationship between blue whales and chl-a concentrations derived from SeaWIFS and *in situ* water sampling, respectively. This may reflect that blue whales aggregate and vocalize in areas with high zooplankton biomass. Similarly, RF indicated that low chl-a is highly important for the production of Z-calls (i.e. the non-feeding call), which could indicate that Antarctic blue whales may be acoustically more active when not feeding.

The results of the RF model provide evidence that blue whale occurrence and acoustic behaviour are sensitive to annual variabilities of major environmental parameters such as chl-a, wind speed, wind direction and stress, SSH and SST. Consequently, blue whales might be vulnerable to either gradual long-term changes or abrupt and persistent short-term changes or variability (i.e. climate change) of those key environmental factors. This might hamper the recovery of this species and lead to habitat loss and local distributional shifts [21,79] as climate variability/change effects may influence the availability and distribution of prey (i.e. Antarctic krill). The Northern Hemisphere blue whale *Balaenoptera musculus* population is considered highly vulnerable to climate change [80]; the vulnerability of Antarctic blue whales to climate change seems to be just as high.

### Conclusions

The RF modelling enabled the explicit interpretation of the complex relationships between blue whales and their environment from our long term acoustic data set. RF performed well for call rates and occurrence modelling, it holds perhaps the greatest promise for acoustic behaviour and occurrence modelling because of its wide versatility that allows it to assume simpler, faster and more interpretable forms with incorporable automatic predictor selection.

Antarctic blue whales showed both latitudinal and longitudinal preferences in the Southern Ocean. Passive acoustic techniques provided useful information on blue whale occurrence and behaviour, however, more direct and continuous acoustic recording of blue whales are needed. Whales preferred relatively colder waters closer to the ice edge with potentially high krill abundances and occurred closer to the ice edge. The year 2007/08 was an environmental anomaly and blue whale call rates responded to the change, suggesting that future changes in environmental conditions have the potential to affect blue whale behaviour and occurrence. The link between environmental conditions and blue whale occurrence and behaviour revealed important biological information essential for improving the management and conservation of this depleted whale species. This study shows the potential influence of long-term systematic environmental change on Antarctic blue whales.
